# Vertebral artery dissection aneurysm in a pediatric patient: A rare case with unusual clinical manifestations, diagnostic, and management challenges

**DOI:** 10.1097/MD.0000000000035906

**Published:** 2023-11-24

**Authors:** Mohammed A. M. Oshi, Mohammed Fahad Aljabri, Saad Alotaibi, Yahea Alzahrani, Jaber Alfaifi, Salma A. S. Abosabie, Sara A. Abosabie, Samaher S. Algethami, Alaa E. Younes, Raid K. Almanjoomi, Bashar Babkour,, Naglaa M. Kamal

**Affiliations:** a Neurology Division, Gaafar Ibnauf Children’s Emergency Hospital, Khartoum, Sudan; b Alhada Armed Forces Hospital, Department of Pediatrics, Neurology Division, Taif, Kingdom of Saudi Arabia; c Department of Radiology, Neuroradiology Section, Alhada Armed Forces Hospital, Taif, Kingdom of Saudi Arabia; d Department of Radiology, Faculty of Medicine, Taif University, Taif, Kingdom of Saudi Arabia; e Department of Child Health, College of Medicine, University of Bisha, Bisha, Saudi Arabia; f Faculty of Medicine, Julius-Maximilians-Universität Würzburg, Bavaria, Germany; g Faculty of Medicine, Charité Universitätsmedizin Berlin, Berlin, Germany; h Taif Children Hospital, Taif, KSA; i Department of Surgery, College of Medicine, Taif University, Taif, Saudi Arabia; j Pediatrics and Pediatric Hepatology, Kasr Alainy Faculty of Medicine, Cairo University, Cairo, Egypt.

**Keywords:** aneurysm, children, computed tomography angiography, magnetic resonance angiography, vertebral artery dissection

## Abstract

Vertebral artery aneurysm is a rare condition with diverse clinical manifestations in pediatric patients. We present the case of a 12-year-old male who presented with diplopia, vomiting, ataxia, and severe headache. Diagnostic evaluation revealed an extracranial vertebral artery dissection with an associated aneurysm at the C3–C4 level. Despite the absence of recurrent ischemic strokes, the aneurysm posed challenges in differentiating the symptoms from other inflammatory demyelinating disorders, particularly internuclear ophthalmoplegia. Diagnosis relied on a thorough history, physical examination, and imaging studies. Magnetic resonance imaging with magnetic resonance angiography confirmed the diagnosis and played a crucial role in assessing the size, location, and extent of the aneurysm. Additionally, the imaging findings helped guide treatment decisions and determine the need for anticoagulation therapy. Regular follow-up imaging was initiated to monitor for late complications and evaluate the effectiveness of the management approach. This case highlights the atypical presentation of vertebral artery aneurysm in a pediatric patient, underscoring the importance of clinical suspicion and the role of advanced imaging techniques in facilitating accurate diagnosis and guiding appropriate management. Prompt diagnosis and optimal utilization of imaging modalities are essential in preventing severe morbidity and mortality. Further research is warranted to enhance our understanding of this condition and refine imaging and management protocols in pediatric population.

**Key Points**

1. Physicians should maintain a high level of suspicion for vertebral artery dissection when encountering cases with unusual or ambiguous headache presentations.
2. This case study emphasizes the diverse array of uncommon clinical manifestations that can accompany vertebral artery dissection, which can complicate treatment decisions.
3. Timely diagnosis of vertebral artery dissection is essential for preventing severe morbidity and mortality. Early detection enables the prompt initiation of appropriate treatment, including long-term anticoagulation therapy.

## 1. Introduction

Acute ischemic stroke (AIS) symptoms often coincide with vertebral artery dissection (VAD) in childhood.^[1]^ It is a rare disease affecting 2.5 out of 100,000 children per year. VAD involves the separation of intimal layers, exposing collagen to the endothelium and activating tissue factors that lead to thrombus formation and propagation.^[1]^ The mechanisms of ischemic stroke in young individuals with craniocervical arterial dissection (CCAD) remain unclear, with intracranial CCAD being more frequent than extracranial CAD, and the vertebral artery being the most commonly affected site.^[2]^

Aneurysmal dilatation, a well-known complication of arterial dissection, frequently occurs in the C1–C2 vertebral circulation in children, resulting from impaired vessel wall integrity and leading to recurrent AIS.^[3,4]^ Risk factors for VAD in children include head and neck injuries, connective tissue disorders, and male gender. Ehlers-Danlos syndrome is the most common connective tissue disorder associated with VAD.^[5,6]^ Minor events such as coughing, vomiting, chiropractic procedures, and blunt trauma are often reported by patients prior to symptom onset.^[5]^

Clinical findings of VAD in children can be challenging to diagnose due to the presence of nystagmus, truncal ataxia, Horner syndrome, sensory disturbance, and internuclear ophthalmoplegia.^[7,8]^ Recent trauma is an important clue for diagnosis, and dissection is more prevalent in the posterior circulation than the anterior circulation in children with AIS.^[9]^ Diffuse headaches are more common in children with AIS, whereas neck pain is more common in adults.^[9]^ Clinical suspicion plays a vital role in diagnosing childhood VAD and AIS.^[10]^

The International Paediatric Stroke Study defines CCAD based on angiographic findings of a double lumen, intimal flap, or pseudoaneurysm, axial T1 fat saturation magnetic resonance imaging (MRI) findings of a bright crescent in the arterial wall, angiographic segmental arterial narrowing in the cervical arteries <6 weeks prior to the findings, cervical/cranial traumatic history, or angiographic segmental narrowing of the vertebral artery at level C2, even without a known traumatic history.^[11,12]^

Spontaneous VAD can occur without significant trauma or after minor trauma, such as head twisting during physical activity or chiropractic manipulation.^[13]^ In one series, minor trauma occurred prior to presentation in 25% of children with vertebral arterial dissection.^[14]^

Diagnostic imaging of the cervical and intracranial vascular system is the cornerstone for confirming CCAD. Available imaging modalities include traditional angiography, computed tomography (CT) angiography, and magnetic resonance angiography (MRA).^[15]^

Treatment approaches for extracranial arterial dissection have become controversial, with some experts advocating for less aggressive therapy such as aspirin, while others employ more invasive techniques such as stenting.^[16]^ Although anticoagulation is the recommended treatment for childhood AIS, there is limited evidence comparing antiplatelet therapy to anticoagulation in both adults and children with CCAD.^[16–18]^ Treatment guidelines vary across international centers, but recent recommendations suggest anticoagulation with agents like unfractionated heparin (UFH), low molecular weight heparin (LMWH), or warfarin for extracranial dissections in children.^[17,18]^ The American Heart Association Scientific Statement suggests starting UFH or LMWH as a bridge to oral anticoagulation for extracranial CCAD in children.^[16]^ Similarly, the American College of Chest Physicians recommends anticoagulant therapy with LMWH or vitamin K antagonists for at least 6 weeks in AIS secondary to dissection, with ongoing treatment based on radiological assessment.^[15,16]^

## 2. Case scenario

A 13-year-old previously healthy boy with excellent school performance presented to the Paediatric Emergency Department with a 7-day history of severe headaches localized in the frontal region. The headaches were accompanied by vomiting and diplopia, but there were no alterations in the level of consciousness, unsteadiness, visual acuity or field defects, abnormal movements, or seizures reported. The patient denied any history of head or neck trauma, except for occasional excessive use of the neck. His past history was non contributary and there was no significant family history of epilepsy, chronic headaches, or stroke.

Upon physical examination, the child was vitally stable, fully conscious, cooperative, well oriented to time, person & place, and of average mood, memory, and intelligence. His weight was 61.6 kg (90th–95th percentile), height was 145 cm (10th–25th percentile), and body mass index was 28.8 kg/m². There were no dysmorphic features or evidence of joint laxity. Ophthalmologic examination revealed internuclear ophthalmoplegia, while visual acuity, visual fields, pupillary light responses, and fundus examination were normal. The patient reported no pain with eye movements. Other than truncal ataxia, all remaining neurological, and systemic examinations were unremarkable.

Different differential diagnoses were considered based on the patient’s clinical presentation varying from serious (intracranial infection, cervical spine fracture, subarachnoid hemorrhage, hemorrhagic stroke, ischemic stroke, vasculitis affecting the vertebrobasilar circulation, etc) to benign causes (cervical strain, migraine headache [vestibular migraine], tension headache, etc).

Work up to evaluate the patient’s condition included complete blood count, renal function tests, electrolyte levels, and liver function tests which were all within normal ranges. In order to exclude infectious causes, a lumbar puncture was performed, which revealed normal opening pressure, white blood cell count, glucose, protein, and cytology. Testing for herpes simplex virus DNA was negative.

**Figure 1.**
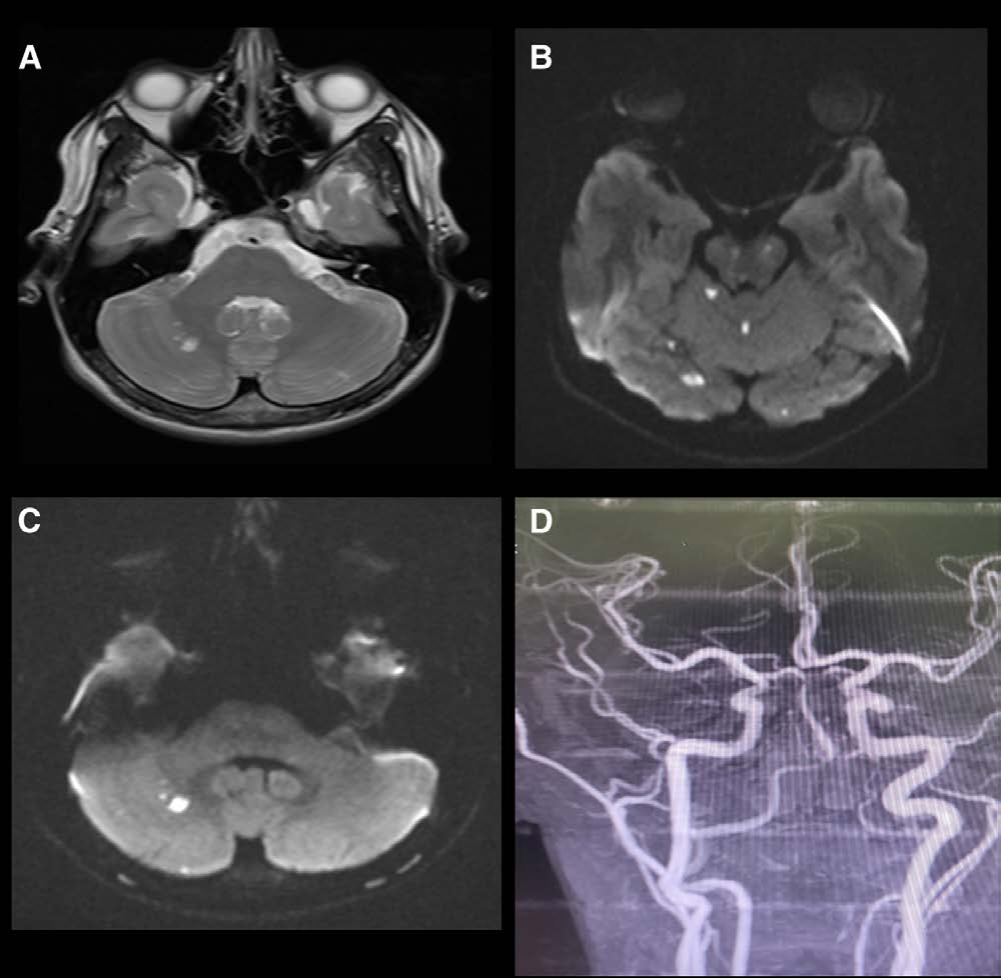
Brain MRI posterior circulation multiple embolic infarcts: (A and B) diffusion weighted images at different levels, (C) T2-weighted image show abnormal T2 discrete hyperintensity foci with diffusion restrictions involving the cerebellum and occipital lobes bilaterally, (D) left vertebral artery narrowing.

**Figure 2.**
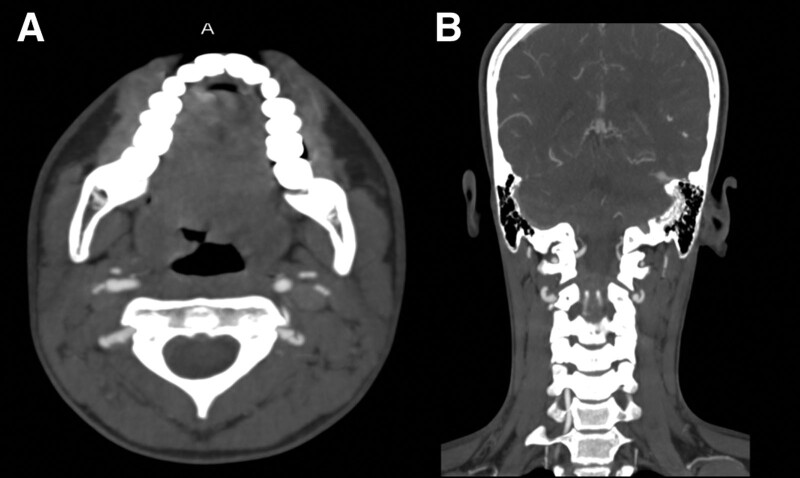
(A and B) Dissecting aneurysm of left vertebral artery: axial and coronal CT angiogram with maximum intensity projection shows small dissecting saccular shaped aneurysm identified at the distal left V3 segment vertebral artery. CT = computed tomography.

To further investigate the patient’s condition, a brain CT scan was performed, which showed no space occupying lesion or other abnormalities. However, brain MRI with contrast revealed the presence of multiple embolic infarctions involving the posterior circulation territory with restriction on diffusion weighted image. MRA of the neck vessels showed narrowing and irregularity of the left vertebral artery at the C3 and C4 levels, suggestive of VAD (Fig. 1A–D). The diagnosis was confirmed by CT angiography which demonstrated evidence of left VAD and a small aneurysmal dilation at the same level (Fig. 2A and B). The spinal cord anatomy appeared normal, and no abnormal signal intensity was observed. Magnetic resonance venography ruled out the presence of venous sinus thrombosis. Thrombophilia screening was performed and did not reveal any abnormalities.

The patient was initially treated with UFH, followed by a switch to aspirin. Within 72 hours of initiating treatment, the patient exhibited significant improvement in diplopia, internuclear ophthalmoplegia, and ataxia, with no evidence of new neurological signs or symptoms. Due to the complexity of the case and the need for further evaluation and management, the patient was referred to a specialized center with interventional radiological facilities. After a comprehensive evaluation, the patient was determined to be ineligible for stent placement. Consequently, he continued treatment with aspirin. Follow-up brain MRI and MRA after 2 months showed no parenchymal abnormalities indicating a favorable response to treatment. The patient reported an improvement in the quality of life. During the most recent clinic visit, the patient remained asymptomatic and gradually returned to his usual activities, with advice to avoid strenuous exercise and dietary advice to reduce weight.

## 3. Discussion

Vertebral artery dissection is a rare condition in children. While connective tissue diseases and trauma are known risk factors for its occurence, they are often absent in most patients (idiopathic), making diagnosis challenging and relying on clinical suspicion alone^[3,4]^ which was similar to our patient who lies in the idiopathic/spontaneous category.

Unlike adults with CCAD, children are more likely to have intracranial involvement, with spontaneous CCAD cases being predominantly intracranial and post-traumatic cases more commonly extracranial.^[19,20]^ While in the presented case, the involvement was extracranial.

In children, VAD can result in aneurysmal dilatation due to the impaired integrity of the vessel wall. This can contribute to recurrent acute ischemic stroke.^[2,4]^ Similar to previous reports our patient, had VAD with an aneurysmal dilation.^[3]^ However, unlike other cases, he did not experience recurrent ischemic strokes.

Our patient was a male which agreed with previous reports which highlighted a male predominance among children with cerebral arterial dissections, which cannot be entirely explained by trauma.^[1,12]^

In VAD, the separation of the vessel wall layers exposes the endothelium to collagen and activates tissue factors, resulting in thrombus formation and propagation. However, our patient did not show evidence of thrombus formation or propagation, which may be attributed to the small size of the aneurysm and the degree of intimal separation.^[8,11]^

Headache is a common clinical symptom in children with VAD, particularly at the C1–C2 level (44%).^[1,12,13]^ Other clinical presentations include altered consciousness (25%), seizures (12.5%), and focal deficits (87.5%).^[13]^ In our case, the patient presented with diplopia, vomiting, and ataxia in addition to a severe headache, possibly due to the dissection being at the C3–C4 level. Initially, differentiating the symptoms as internuclear ophthalmoplegia, associated with inflammatory demyelinating disorders, posed a challenge.

The diagnosis of CCAD in children relies on a thorough history and physical examination, especially for those presenting with transient ischemic attack or AIS.^[15,18]^ The American Heart Association Stroke Council has published evidence-based recommendations for the prevention and management of ischemic strokes caused by various conditions, including CCAD. These recommendations emphasize the need for evaluation and management protocols in children, considering the risks and benefits of different imaging techniques and anticoagulation therapies.^[16]^ MRI with MRA is recommended as the first-line imaging study for suspected CCAD in children.^[15,18]^ Children with VAD aneurysms were more likely to present with stroke at a younger age and have recurrent strokes compared to those without aneurysms. Initial diagnostic evaluations in children with stroke often miss VAD aneurysms, underscoring the importance of comprehensive, regular follow-up.^[7,9]^

The treatment of CCAD in children is challenging and differs for intracranial and extracranial cases. In extracranial cases, anticoagulation therapy is commonly used for 6 weeks to 6 months in patients with transient ischemic attack or arterial ischemic stroke.^[7,15,21]^ Antithrombotic treatment is commonly administered to children with CCAD, regardless of the etiology.^[13,15,21]^ There was no significant difference observed by between patients receiving antiplatelet or anticoagulation therapy at the time of diagnosis.^[7,9]^

Regarding management outcomes, complete recovery was observed in 43% of cases, mild to moderate deficits in 44%, and severe deficits in 13%. Regular follow-up is crucial to monitor for late complications, such as recurrent stroke, and to guide long-term management and prevention of recurrence.^[7,9]^

## 4. Conclusions

It is important for healthcare providers to remain vigilant and consider the possibility of VAD in patients with atypical or unclear presentations of headaches. The case discussed in this report underscores the wide range of unusual clinical symptoms associated with VAD, which can make treatment decisions challenging. Early and accurate diagnosis of VAD is crucial to prevent serious complications and potential fatalities. Timely identification allows for the initiation of appropriate treatment, including long-term anticoagulation therapy. Regular follow-up is necessary to monitor for any potential complications, prevent recurrence, and manage the condition effectively.

## Acknowledgments

We express our thanks to the patient and his family for their contribution in the current study.

## Author contributions

**Conceptualization:** Mohammed A. M. Oshi, Mohammed Fahad Aljabri.

**Data curation:** Mohammed A. M. Oshi, Mohammed Fahad Aljabri, Alaa E. Younes, Salma A. S. Abosabie, Sara A. Abosabie, Samaher S. Algethami, Raid K. Almanjoomi, Naglaa M. Kamal.

**Investigation:** Mohammed A. M. Oshi, Mohammed Fahad Aljabri, Saad Alotaibi, Yahea A. L. Zahrani, Bashar Babkour.

**Methodology:** Mohammed A. M. Oshi, Saad Alotaibi, Yahea A. L. Zahrani.

**Project administration:** Mohammed A. M. Oshi, Saad Alotaibi, Yahea A. L. Zahrani.

**Supervision:** Saad Alotaibi, Yahea A. L. Zahrani.

**Validation:** Saad Alotaibi, Yahea A. L. Zahrani.

**Writing – original draft:** Mohammed A. M. Oshi, Jaber Alfaifi, Salma A. S. Abosabie, Sara A. Abosabie, Samaher S. Algethami, Naglaa M. Kamal.

**Writing – review & editing:** Mohammed A. M. Oshi, Jaber Alfaifi, Salma A. S. Abosabie, Sara A. Abosabie, Naglaa M. Kamal.
